# High Homocysteine and Blood Pressure Related to Poor Outcome of Acute Ischemia Stroke in Chinese Population

**DOI:** 10.1371/journal.pone.0107498

**Published:** 2014-09-29

**Authors:** Chongke Zhong, Liying Lv, Changjiang Liu, Liang Zhao, Mo Zhou, Wenjie Sun, Tan Xu, Weijun Tong

**Affiliations:** 1 Department of Epidemiology, School of Public Health, Medical College of Soochow University, Suzhou, Jiangsu, China; 2 Department of Neurology, Xinganmeng People's Hospital, Xinganmeng, Inner Mongolia, China; 3 Department of Neurology, Fuxin Center Hospital, Fuxin, LiaoNing, China; 4 Department of Neurology, Affiliated Hospital of Chengde Medical University, Chengde, HeBei, China; 5 School of Food Science, Guangdong Pharmaceutical University, Zhongshan, China; 6 School of Public Health and Tropical Medicine, Tulane University, New Orleans, Louisiana, United States of America; 7 Department of Epidemiology, School of Public Health and Tropical Medicine, Tulane University, New Orleans, New Orleans, Louisiana, United States of America; University of Modena and Reggio Emilia, Italy

## Abstract

**Objectives:**

To assess the association between plasma homocysteine (Hcy), blood pressure (BP) and poor outcome at hospital discharge among acute ischemic stroke patients, and if high Hcy increases the risk of poor outcome based on high BP status in a northern Chinese population.

**Methods:**

Between June 1, 2009 and May 31, 2013, a total of 3695 acute ischemic stroke patients were recruited from three hospitals in northern Chinese cities. Demographic characteristics, lifestyle risk factors, medical history, and other clinical characteristics were recorded for all subjects. Poor outcome was defined as a discharge modified Rankin Scale (mRS) score ≥3 or death. The association between homocysteine concentration, admission blood pressure, and risk of poor outcome following acute ischemic stroke was analyzed by using multivariate non-conditional logistic regression models.

**Results:**

Compared with those in the lowest quartile of Hcy concentration in a multivariate-adjusted model, those in the highest quartile of Hcy concentration had increased risk of poor outcome after acute ischemic stroke, (OR = 1.33, *P*<0.05). The dose-response relationship between Hcy concentration and risk of poor outcome was statistically significant (p-value for trend  = 0.027). High BP was significantly associated with poor outcome following acute ischemic stroke (adjusted OR = 1.44, 95%CI, 1.19–1.74). Compared with non-high BP with nhHcy, in a multivariate-adjusted model, the ORs (95% CI) of non-high BP with hHcy, high BP with nhHcy, and high BP with hHcy to poor outcome were 1.14 (0.85–1.53), 1.37 (1.03–1.84) and 1.70 (1.29–2.34), respectively.

**Conclusion:**

The present study suggested that high plasma Hcy and blood pressure were independent risk factors for prognosis of acute ischemic stroke, and hHcy may further increase the risk of poor outcome among patients with high blood pressure. Additionally, the results indicate that high Hcy with high BP may cause increased susceptibility to poor outcome among acute ischemic stroke patients in a northern Chinese population.

## Introduction

Stroke is the second most common cause of death and the leading cause of serious, long-term disability worldwide [1]. The burden of stroke is high and growing in economically developing countries [2]. In China, stroke accounts for 21.6% of total mortality in males and 20.8% of total mortality in females [3]. The total incidence of stroke is projected to increase considerably over the next two decades [4].

Homocysteine (Hcy), a sulphur-containing amino acid, was hypothesized to affect atherosclerotic processes as early as 1969 [5]. Since that time, numerous epidemiological studies have demonstrated a positive association between Hcy level and stroke incidence [6]–[9]. A meta-analysis of prospective studies supports the possibility that the association between plasma Hcy and stroke may be causal: a 5 µmol/l Hcy elevation causes a 59% increase in stroke risk, while a 3 µmol/l Hcy reduction leads to a 24% decrease in stroke risk [9]. Previous research has found that elevated Hcy is associated with poor outcome among acute ischemic stroke patients [10]–[12], but inconsistent results still exist [13]–[16]. A study in a Korean population, after a 12-month follow-up, shows that plasma Hcy levels have no value as predictors of functional outcome [13].

Epidemiological studies have documented that high blood pressure (BP) is the most important modifiable risk factor for the incidence of stroke [17], [18]. In China, hypertensive patients usually have high homocysteine (hHcy) concentration levels and the proportion is about 75%. A large sample of epidemiological research demonstrated that the incidence of cardiovascular events in hypertensive patients with high Hcy is about 5 times higher than in patients with hypertension alone, and about 25–30 times higher than in those without hypertension [19]. And a nationwide study showed elevated total plasma homocysteine and hypertension had a multiplicative effect on the odds of prevalent stroke [20]. Few studies to date have examined the cumulative effect of high BP at admission and Hcy concentration on poor outcome among stroke patients, especially in a northern Chinese population.

Thus, we sought to assess the relationship between high plasma homocysteine, blood pressure and poor discharge outcome among acute ischemic stroke patients. We also examined if hHcy may increase the risk of poor outcome based on high BP status in a northern Chinese population.

## Methods

### Study participants

From June 1, 2009 to May 31, 2013, we recruited adult patients with a clinical diagnosis of acute ischemic stroke from three hospitals in northern cities in China (the People's Hospital of XingAnMeng in Inner Mongolia, the Center Hospital of FuXin in LiaoNing province and ChengDe Medical College Affiliated Hospital in HeBei province). All participants were recruited within 7 days of symptom onset. Exclusion criteria were: (1) lack of plasma homocysteine concentration measurement; (2) lack of blood pressure data; (3) presence of illnesses including renal and kidney disease, malignancy, autoimmune disease, and hypothyroidism; and (4) inability to comply with a magnetic resonance examination.

### Ethics Statement

This study was approved by the Soochow University Ethics Committee. Written informed consent was obtained from all study participants.

### Data Collection

Demographic characteristics, lifestyle risk factors, medical history, clinical laboratory tests and imaging data (CT and MRI) were recorded within the first 24 h after admission to hospital by a face to face interview with patients or their family members (if patients were not able to communicate). All information was obtained using a standard questionnaire administered by trained staff. Cigarette smoking was defined as having smoked at least 1 cigarette per day for 1 year or more. The amount and type of alcohol consumed during the past year were collected. Alcohol consumption was defined as consuming one or more alcoholic drink per day during the last year. Blood pressure (BP) was measured in the first 72 h (one measurement every 8 h) after admission while the participants were in the supine position using a standard mercury sphygmomanometer according to a standard protocol. The first and fifth Korotkoff sounds were recorded as systolic (SBP) and diastolic BP (DBP), respectively. High admission BP was defined as SBP ≥140 mmHg and/or DBP ≥90 mmHg. History of hypertension was defined that patients have hypertension prior to admission to hospital, according to the question “have you received a diagnosis of hypertension from any doctor or hospital before this onset of stroke”, or use of antihypertensive medication, or SBP ≥140 mmHg and/or DBP ≥90 mmHg.

Hcy, total cholesterol, high-density lipoprotein cholesterol, low-density lipoprotein cholesterol and triglycerides were analyzed on a Beckman Synchron CX5 Delta Clinical System (Beckman Coulter, Inc., Fullerton, California, USA) using commercial reagents [21]. Plasma glucose was measured by a modified hexokinase enzymatic method [22]. The criterion of dyslipidemia was as follows: total cholesterol ≥6.22 mmol/l or triglyceride ≥2.26 mmol/l or LDL cholesterol ≥4.14 mmol/l or HDL cholesterol <1.04 mmol/l. Hyperglycemia was defined as fasting plasma glucose ≥6.1 mmol/l. High Hcy (hHcy) was defined as Hcy>15 umol/l, and nhHcy was Hcy ≤15 umol/l [23], [24].

Acute ischemic stroke was confirmed by imaging (CT scan or MRI) according to the acute ischemic stroke diagnosis and treatment guidelines in China. Study outcome was evaluated using the modified Rankin Scale (mRS) obtained at hospital discharge, and poor outcome was defined as a mRS score ≥3 or death. Scores on the modified Rankin Scale range from 0 to 6, with a score of 0 indicating no symptoms; a score of 5 indicating severe disability (i.e., bedridden, incontinent, or requiring constant nursing care and attention); and a score of 6 indicating death [25].

### Statistical analysis

Statistical analysis was conducted using SAS statistical software (version 9.1, Cary, North Carolina, USA). Patients were divided into two groups according to study outcome: mRS <3 and mRS ≥3 or death. According to blood pressure and concentration level of Hcy, participants were divided into four groups: non-high BP with non-hHcy (nhHcy), non-high BP with hHcy, high BP with nhHcy, and high BP with hHcy. Baseline characteristics of continuous variables were compared in 2 or 4 subgroups using Student's t-test or ANOVA. Categorical variables were expressed as percentage and compared between groups using Chi-square test. Plasma Hcy concentration was divided into four quartiles (<11.9 µmol/l, 11.9–16.1 µmol/l, 16.1–23.3 µmol/l, ≥23.3 µmol/l; <11.9 µmol/l is the lowest quartile and ≥23.3 µmol/l is the highest quartile). Univariate and multivariate non-conditional logistic regression models were used to assess the association between Hcy, blood pressure and discharge outcome among acute ischemic stroke patients. Odd ratios (ORs) and 95% confidence intervals (95% CIs) were used to evaluate the risk of poor outcome after adjusting for important confounding factors. Multivariable interaction models were used to evaluate the interaction of Hcy and blood pressure with poor outcome among acute ischemic stroke patients. All P values were 2-tailed, and a significance level of 0.05 was used.

## Results

A total of 3695 patients (2381 males and 1314 females) were included in this study. The mean age of the participants was 62.40±11.45 years. Table 1 shows baseline characteristics at admission in patients with good outcome (mRS  = 0,1,2) and poor outcome (mRS  = 3,4,5 or dead). Age, high BP percentage, hospitalized days and SBP were higher in the poor outcome group whereas onset to hospital admission time, TG, TC, LDL cholesterol and proportion of dyslipidemia were higher in the good outcome group. There were no significant differences in Hcy and other variables between the two groups.

**Table 1:** Baseline characteristics of acute ischemic stroke patients with good outcome or poor outcome.

	Good outcome (mRS = 0,1,2)	Poor outcome (mRS = 3,4,5 or dead)	P-value
N	3105	590	
Age	62.14±11.27	63.77±12.27	0.006
Male no,%	2009,64.70	372,63.05	0.442
Days hospitalized (days)	13.21±7.71	16.94±12.00	<0.001
Onset to hospital admission time (hours)	48(18,106)	48(10,96)	0.001
Smoker no,%	1184,38.13	226,38.31	0.937
Drinker no,%	980,31.56	170,28.81	0.186
Admission SBP (mmHg)	145.61±27.66	148.55±23.57	0.007
Admission DBP (mmHg)	88.33±13.15	89.36±13.11	0.081
High BP no,%	1593,51.30	360,61.02	<0.001
History of Hypertension no,%	2054,66.15	398,67.46	0.538
History of Hyperglycemia no,%	615,19.81	115,19.49	0.860
History of coronary heart disease no,%	406,13.08	94,15.93	0.063
History of atrial fibrillation, no,%	50,1.61	16,2.71	0.064
Glucose (mmol/l)	6.75±3.63	6.63±3.09	0.435
Hyperglycemia no,%	1285,41.38	244,41.36	0.989
TG (mmol/l)	1.79±1.39	1.53±1.62	<0.001
TC (mmol/l)	4.84±1.79	4.60±1.19	<0.001
LDL cholesterol (mmol/l)	3.14±0.87	3.02±0.97	0.007
HDL cholesterol (mmol/l)	1.12±0.30	1.10±0.28	0.135
Dyslipidemia no,%	1766,56.88	305,51.69	0.020
Hcy (umol/l)	20.40±15.31	21.78±17.23	0.071

All values are expressed with mean±standard deviation unless otherwise noted. SBP, systolic blood pressure; DBP, diastolic blood pressure; BP, blood pressure; TG, triglycerides; TC, total cholesterol; LDL, low-density lipoprotein; HDL, high-density lipoprotein; Hcy, homocysteine.

Table 2 shows that there was a significant association between Hcy concentration and risk of poor outcome. Compared with patients in the lowest quartile of Hcy concentration, those in the highest quartile faced an increased risk of poor outcome (OR = 1.29, *P*<0.05). After adjustment for age, gender, days hospitalized, onset to hospital admission time, smoking, drinking, history of hypertension, history of hyperglycemia, history of coronary heart disease, history of atrial fibrillation, high BP, hyperglycemia, and dyslipidemia, those in the highest quartile still had an increased risk of poor outcome (OR = 1.33, *P*<0.05). The dose-response relationship between Hcy concentration and risk of poor outcome remained significant after adjustment for other covariates (p-value for trend  = 0.027).

**Table 2:** Odd ratio (OR) and 95% confidence interval (CI) of poor outcome by quartiles of homocysteine level among acute ischemia stroke patients.

			Unadjusted			Multivariable Adjusted*		
Hcy umol/l	Participants N	Patients with poor outcome n,%	OR	95%CI	P-value	OR	95%CI	P-value
≤11.9	943	136,14.42	1.00	-		1.00	-	
11.9∼16.1	914	136,14.88	1.04	0.80∼1.34	0.780	1.07	0.82∼1.40	0.628
16.1∼23.3	924	155,16.77	1.20	0.93∼1.54	0.162	1.19	0.91∼1.55	0.218
>23.3	914	163,17.83	1.29	1.01∼1.65	0.046	1.33	1.02∼1.75	0.036
P-value for trend				0.025			0.027	

*Multivariable model adjusted for age, gender, days hospitalized, onset to hospital admission time, smoking, drinking, history of hypertension, history of hyperglycemia, history of coronary heart disease, history of atrial fibrillation, high BP, hyperglycemia, and dyslipidemia.

As shown in Table 3, ORs of poor outcome were significant in patients with an onset to hospital admission time>96 hours, those with a history of hypertension, and those with dyslipidemia. The ORs (95%CIs) were 1.32 (1.04–1.68), 1.17 (1.02–1.33) and 1.23 (1.06–1.44), respectively.

**Table 3:** Sub-group analysis on the association between plasma homocystein and poor outcome among acute ischemic stroke patients.

	NO. of participants		Poor outcome n,%		
Subgroup	nhHcy	hHcy	nhHcy	hHcy	OR(95%CI)
Age, y					
≤62	967	957	131,13.6	144,15.0	1.06(0.93–1.22)
>62	685	1086	112,16.4	203,18.7	1.11(0.94,1.30)
Gender					
Male	846	1535	122,14.4	250,16.3	1.10(0.94–1.29)
Female	806	508	121,15.0	97,19.1	1.13(0.99–1.28)
Days hospitalized, d					
≤13	862	1115	95,11.0	148,13.3	1.13(0.96–1.34)
>13	790	928	148,18.7	199,21.4	1.10(0.96–1.26)
Onset to hospital admission time, h					
≤24	719	888	113,15.7	164,18.5	1.12(0.96–1.30)
24–96	467	543	82,17.6	87,16.0	0.95(0.80–1.12)
>96	466	612	48,10.3	94,15.4	1.32(1.04–1.68)
Smoker					
No	1137	1148	169,14.9	195,17.0	1.09(0.96–1.22)
Yes	515	895	74,14.4	152,17.0	1.14(0.93–1.39)
Drinker					
No	1226	1319	191,15.6	229, 17.4	1.07(0.96–1.20)
Yes	426	724	52,12.2	118, 16.3	1.25(0.98–1.59)
High BP					
No	788	954	98,12.4	132, 13.8	1.07(0.91–1.26)
Yes	864	1089	145,16.8	215, 19.7	1.21(0.98–1.29)
History of Hypertension					
No	585	658	91,15.6	101, 15.3	0.99(0.84–1.17)
Yes	1067	1385	152,14.2	246, 17.8	1.17(1.02–1.33)
Hyperglycemia					
No	885	1281	127,14.4	219, 17.1	1.14(0.98–1.32)
Yes	767	762	116,15.1	128,16.8	1.07(0.92–1.23)
Dyslipidemia					
No	716	908	128.17.9	157,17.3	0.98(0.85–1.13)
Yes	936	1135	115,12.3	190,16.7	1.23(1.06–1.44)

hHcy was defined as Hcy>15 umol/l, and nhHcy was Hcy ≤15 umol/l.

We also analyzed the independent effect of admission blood pressure on poor outcome after acute ischemic stroke. The multivariable-adjusted OR (95% CI) of poor outcome was 1.44 (1.19–1.74). Table 4 summarizes the baseline characteristics of participants according to blood pressure and level of Hcy. Conventional risk factors, such as gender, days hospitalized, onset to hospital admission time, smoking, drinking, SBP, DBP, history of hypertension, history of hyperglycemia, history of coronary heart disease, history of atrial fibrillation, hyperglycemia, TG, TC and HDL cholesterol, were significantly different among the 4 groups. Namely, smoking, drinking, history of hyperglycemia, history of coronary heart disease, hyperglycemia, and TG were lower in those with hHcy in either high BP group. Hospitalized days, admission SBP, DBP, history of hypertension, diabetes mellitus percentage, TC, LDL cholesterol, and HDL cholesterol were higher in those with hHcy or nhHcy and high BP.

**Table 4:** Baseline characteristics of participants according to blood pressure and level of Hcy.

	Non-high BP nhHcy	Non-high BP hHcy	High BP nhHcy	High BP hHcy	P-value
N	1332	410	1449	504	
Age	62.4±11.4	63.4±12.3	62.3±11.2	62.2±11.6	0.323
Male no,%	806,60.51	342,83.41	848,58.52	385,76.39	<0.001
Days hospitalized (days)	13.5±8.2	12.8±6.2	14.4±9.8	13.7±7.6	0.001
Onset to hospital admission time (hours)	48(20,128)	56(24,120)	48(12,120)	48(10,124)	<0.001
Smoker no,%	477,35.81	209,50.98	476,32.85	248,49.21	<0.001
Drinker no,%	369,27.70	160,39.02	418,28.85	203,40.28	<0.001
Admission SBP (mmHg)	128.2±11.5	127.9±10.6	161.9±29.4	162.7±18.2	<0.001
Admission DBP (mmHg)	80.0±8.00	79.9±8.08	95.5±11.9	97.7±12.9	<0.001
History of Hypertension no,%	701,52.63	236,57.56	1133,78.19	382,75.79	<0.001
History of Hyperglycemia no,%	287,21.55	51,12.44	322,22.22	70,13.89	<0.001
History of coronary heart disease no,%	190,14.26	55,13.41	207,14.29	48,9.52	0.041
History of atrial fibrillation no,%	27,2.03	12,2.93	25,1.73	2,0.40	0.029
Glucose (mmol/l)	6.69±4.21	6.48±4.33	6.90±2.88	6.52±2.48	0.058
Hypertension no,%	514,38.59	141,34.39	673,46.45	201,39.88	<0.001
Tg (mmol/l)	1.75±1.37	1.63±1.11	1.82±1.66	1.61±1.00	0.010
Tc (mmol/l)	4.75±1.16	4.62±1.06	4.87±1.83	4.89±2.70	0.021
LDL cholesterol (mmol/l)	3.09±0.86	3.06±0.86	3.15±0.90	3.16±0.93	0.147
HDL cholesterol (mmol/l)	1.11±0.28	1.06±0.25	1.12±0.31	1.12±0.32	0.007
Dyslipidemia no,%	733,55.03	235,57.32	828,57.14	275,54.56	0.576

All values are expressed with mean±standard deviation unless otherwise noted. SBP, systolic blood pressure; DBP, diastolic blood pressure; Tg, triglycerides; Tc, total cholesterol; LDL, low-density lipoprotein; HDL, high-density lipoprotein; Hcy, homocysteine.

As shown in table 5, compared to those with non-high BP and nhHcy, those with high BP in the nhHcy and hHcy groups were at higher risk of poor outcome following acute ischemic stroke: ORs (95%CIs) were 1.42 (1.08–1.87) and 1.73 (1.34–2.24), respectively. After adjustment for other confounding factors, the ORs (95%CIs) of non-high BP with hHcy, high BP with nhHcy, and high BP with hHcy groups were 1.14 (0.85–1.53), 1.37 (1.03–1.84), and 1.70 (1.29–2.34), respectively, compared with the non-high BP with nhHcy group. High BP with hHcy was associated with a higher risk of poor outcome. No significant interaction was detected between high BP and hHcy on poor outcome following acute ischemic stroke (*P* = 0.68).

**Table 5:** Unadjusted and multivariable-adjusted odd ratios for poor outcome according to blood pressure and level of Hcy.

			Unadjusted			Multivariable Adjusted*		
	Participants N	Patients with poor outcome n,%	OR	95%CI	P-value	OR	95%CI	P-value
Non-high BP/nhHcy	788	98,12.44	1.00	-		1.00	-	
Non-high BP/hHcy	954	132,13.84	1.13	0.85–1.50	0.392	1.14	0.85–1.53	0.384
High BP/nhHcy	864	145,16.78	1.42	1.08–1.87	0.013	1.37	1.03–1.84	0.032
High BP/hHcy	1089	215,19.74	1.73	1.34–2.24	<0.001	1.70	1.29–2.34	<0.001

*Multivariable model adjusted for age, gender, days hospitalized, onset to hospital admission time, smoking, drinking, history of hypertension, history of hyperglycemia, history of coronary heart disease, history of atrial fibrillation, hyperglycemia, and dyslipidemia.

## Discussion

In this study, we found that there was a positive and significant association between plasma Hcy concentration and poor discharge outcome among acute ischemic stroke patients. Additionally, the results indicated that blood pressure was an independent risk factor for poor outcome among acute ischemic stroke in this study. Meanwhile, those with high BP and nhHcy or with high BP and hHcy were at a significantly higher risk of poor outcome compared to those with non-high BP and nhHcy. Participants with high BP and hHcy were at the highest risk in this population. To the best of our knowledge, this is the first multi-center study conducted to assess the cumulative effect of high admission BP and Hcy concentration on poor discharge outcome following acute ischemia stroke in three northern cities of China. Compared with previous studies in which Hcy and blood pressure were each shown separately to predict prognosis of stroke [12], [13], [26], the novelty of our study lies in its combination of these two factors to form a new variable which tests the cumulative effect on the risk of poor outcome.

Several epidemiological studies indicated that elevated Hcy concentration is an important risk factor for short-term and long-term poor outcome following acute ischemia stroke [11]–[13], [27]. A prospective study by Tu et al. suggested that Hcy is an independent predictor of short-term outcome and mortality after acute ischemic stroke: the ORs (95%CIs) are 1.24 (1.10–1.39) and 1.14 (1.03–1.27), respectively [10]. Naess et al.'s cohort study indicated that there is an association between Hcy level and subsequent mortality in young adults in the years of following an ischemic stroke, even adjusting for traditional risk factors [11]. Meanwhile, Pniewski et al. assessed the influence of Hcy level on the prognosis of stroke at onset and demonstrated that mild hyperhomocysteinemia (over 15 µmol/l) could increase the risk of poor recovery for stroke [27]. These conclusions above were consistent with ours.

Some experimental studies indicate that Hcy may cause vascular inflammation and oxidative stress, damage endothelial cells, inhibit endothelium dependent relaxation, and enhance thrombogenicity [28]–[30]. Both minor genetic abnormalities and nutritional deficiencies of B vitamins, such as folic acid, involved in metabolism of methionine could lead to increased Hcy concentration. Wang et al.'s meta-analysis indicated that folic supplementation significantly reduced the risk of stroke in primary prevention by 18% (RR 0.82, 95% CI 0.68–1.00; *P* = 0.045) [31].

Increased BP in the acute stage of ischemic stroke may be associated with poor functional outcome because there is augmentation of cerebral edema, hemorrhagic transformation, or stroke recurrence [26], [32], [33]. The International Stroke Trial (IST) demonstrated both high blood pressure and low blood pressure were independent prognostic factors for poor outcome [34]. Our study indicated high BP was an independent risk factor for poor discharge outcome of acute ischemic stroke. Homocysteine may elevate blood pressure by causing arterial stiffness due to impaired vascular endothelial integrity and/or by reducing the efficiency of vasodilation [35]. The National Health and Nutrition Examination Survey (NHANES), a nationally representative cross-sectional sample of the US population, showed that individuals with a combination of elevated total Hcy and hypertension were substantially more likely to have prevalent stroke compared to individuals without either condition (OR 12.02, 95%CI 6.36–22.73 for men and OR 17.34, 95%CI 10.49–28.64 for women), and the survey also indicated that elevated total Hcy and hypertension had a multiplicative effect on the odds of prevalent stroke [20]. However, the cumulative effect of high BP and Hcy concentration on the risk of poor outcome is still unclear. Our data indicated that high BP with hHcy resulted in a higher risk for poor discharge outcome in this population, with a 1.70-fold increased risk compared with non-high BP with nhHcy. Elevated Hcy with high BP seems to increase the risk of poor outcome among acute ischemic stroke patients. The coexistence of elevated Hcy and high BP is a notable issue and suggests that lowering Hcy concentration may improve the prognosis of patients with acute ischemic stroke beyond antihypertensive treatment alone.

In the present study, the mean age of the participants was 62.40±11.45 years, which is lower than that in western countries [36], [37]. The mean age of people with stroke is increasing worldwide, possibly due to an ageing population; however, a worrisome trend of increasing stroke incidence in young adults has been reported for some countries, especially in low-income developing nations [36]. This is largely attributable to the worldwide epidemic of hypertension and the increasing prevalence of smoking and other cardiovascular risk factors in young adults. Therefore, prevention is considered an effective strategy for both primary and secondary prevention of stroke. An overall healthy lifestyle, which includes not smoking, following a well-balanced diet, exercising regularly, drinking moderately, and controlling weight, may be effective in lowering blood pressure and other risk factors.

The potential limitations of this paper merit consideration. Firstly, blood pressure and plasma Hcy concentration were recorded only once at baseline, and we have no data on possible changes in blood pressure and Hcy concentration during the follow-up. Future studies should examine the association between blood pressure and Hcy concentration changes and prognosis of acute ischemic stroke. Secondly, all participants were recruited upon hospital admission and within 7 days of symptom onset, and due to this possible delay of up to 7 days since symptom onset, some patients' Hcy concentration and blood pressure at admission may not accurately reflect the levels of Hcy and blood pressure at stroke onset. In addition, the follow-up period of our study is relatively short, which prevented an evaluation of the long-term effects of plasma Hcy on acute ischemic stroke outcomes.

In conclusion, our study suggested that high Hcy and blood pressure were independent risk factors for prognosis of acute ischemic stroke, and high Hcy may further increase the risk of poor outcome among patients with high blood pressure. Additionally, the results indicated that high Hcy with high BP may cause increased susceptibility to poor outcome among acute ischemic stroke patients in a northern Chinese population.
